# Trial protocol: a clustered, randomised, longitudinal, type 2 translational trial of alcohol consumption and alcohol-related harm among adolescents in Australia

**DOI:** 10.1186/s12889-018-5452-3

**Published:** 2018-04-27

**Authors:** B. Rowland, C. Abraham, R. Carter, J. Abimanyi-Ochom, A. B. Kelly, P. Kremer, J. W. Williams, R. Smith, J. K. Hall, D. Wagner, H. Renner, T. Hosseini, A. Osborn, M. Mohebbi, J. W. Toumbourou

**Affiliations:** 1 Deakin University, Geelong, Victoria Australia; 2 School of Psychology, Centre for Social and Early Emotional Development, Faculty of Health, Geelong, Australia; 3 Institute of Health Research, University of Exeter Medical School St Luke’s Campus, Exeter, EX1 2LU UK; 4 Deakin University, Burwood, Victoria Australia; 5 School of Health and Social Development, Faculty of Health, Geelong, Australia; 6 Centre for Youth Substance Abuse Research, The University of Queensland, Brisbane, Australia; 7 School of Exercise and Nutrition Sciences, Faculty of Health, Geelong, Australia; 8 Murdoch Children Research Institute, The Royal Children’s Hospital, Road Parkville Victoria, Flemington, Australia; 9 Biostatistics unit, Faculty of Health, Melbourne, Australia

## Abstract

**Background:**

This cluster randomised control trial is designed to evaluate whether the Communities That Care intervention (CTC) is effective in reducing the proportion of secondary school age adolescents who use alcohol before the Australian legal purchasing age of 18 years. Secondary outcomes are other substance use and antisocial behaviours. Long term economic benefits of reduced alcohol use by adolescents for the community will also be assessed.

**Methods:**

Fourteen communities and 14 other non-contiguous communities will be matched on socioeconomic status (SES), location, and size. One of each pair will be randomly allocated to the intervention in three Australian states (Victoria, Queensland and Western Australia). A longitudinal survey will recruit grade 8 and 10 students (M = 15 years old, *N* = 3500) in 2017 and conduct follow-up surveys in 2019 and 2021 (M = 19 years old). Municipal youth populations will also be monitored for trends in alcohol-harms using hospital and police administrative data.

**Discussion:**

Community-led interventions that systematically and strategically implement evidence-based programs have been shown to be effective in producing population-level behaviour change, including reduced alcohol and drug use. We expect that the study will be associated with significant effects on alcohol use amongst adolescents because interventions adopted within communities will be based on evidence-based practices and target specific problems identified from surveys conducted within each community.

**Trial registration:**

The trial was retrospectively registered in September, 2017 (ACTRN12616001276448), as communities were selected prior to trial registration; however, participants were recruited after registration. Findings will be disseminated in peer-review journals and community fora.

## Background

Community-led interventions that systematically and strategically implement evidence-based programs have been shown to be effective in producing population-level behaviour change, including reduced alcohol and drug use. Australian guidelines recommend that adolescents should not consume alcohol before they are 18 years but 44% do, with 34% reporting use in the last 30 days [1]. Community interventions such as the “Communities That Care” prevention framework have the potential to reduce these adolescent alcohol consumption (CTC; [2–4]. The CTC framework can be used to mobilise community stakeholders and organise them to work strategically to prevent the development of harmful behaviour patterns. Communities are supported to utilise local data to identify priorities and then to plan the implementation of interventions that have been found to be effective in previous evaluations.

Large community trials in the United States of America (USA) reveal that CTC can increase community implementation of evidence-based prevention interventions and reduce health-risk behaviour patterns among teenagers, resulting in reductions of between 15% - 25% in the prevalence of youth problems [2, 4]. Economic evaluations also indicate that CTC is worthwhile investment. Conservative estimates suggest CTC can produce a return on investment of approximately US$10.23 for every dollar [5, 6]. Over ten years, pilot work developing CTC in Australia has facilitated recruitment and training of community coalitions to plan and implement evidence-based programs. This work has demonstrated feasibility of CTC in Australia. Pilot studies have shown the CTC approach has been successfully implemented with fidelity and has been associated with reductions in teenage alcohol and drug use in line with those achieved in the USA (i.e. 15%) [7]. This study will employ a Type II translational randomised control trial to evaluate the effectiveness of CTC in Australia.

The primary outcome will be the proportion of secondary school age adolescents who use alcohol before the Australian legal purchasing age of 18 years. Based on pilot work done in Australia and CTC trial work done in the USA, we hypothesise that: compared to control communities, participants in intervention communities will maintain at least a 15% lower rate of frequent (last 30 days) youth alcohol use throughout the secondary school age period (primary outcome).

Secondary outcomes are other substance use and antisocial behaviours and the long-term economic benefits of reduced alcohol use by adolescents for the community. Overall, the research objectives for the trial are as follows:

1. To deliver evidence-based alcohol-use prevention interventions in secondary schools as well as evidence-based community interventions targeting parents and teenagers, targeting teenagers aged 12 to 18.
2. To evaluate the intervention using a longitudinal cohort survey and an analysis of archival and prospective health, crime and education records (routine service data).
3. To conduct a process evaluation to assess intervention implementation and mechanisms of action.
4. To estimate the economic benefits of reducing population rates of secondary school age alcohol use.
5. To report on the cost-benefit of the intervention from a number of perspectives, using both trial-based and modelled economic methods.

## Methods

### The communities that care intervention

There are five phases to the CTC framework; these provide structure and benchmarking intervention implementation, monitoring and evaluation steps and processes (see https://www.communitiesthatcare.org.au/5-phases-ctc). In intervention communities, a lead agency (e.g. a Local Council) will be identified and required to lead the community through the phases, receiving a small grant on initiation of each phase. Standardised training sessions for each of the five phases will be delivered and a trained community relations officer appointed in each of the intervention communities.

Three strategies will be employed in intervention communities.

Brief Communication in Schools have been shown to be effective in reducing alcohol consumption [8]. This strategy targets teenagers and their parents and will be delivered during 3 school classroom lessons, promoting the following three messages:

1. Based on scientific evidence, the National Health and Medical Research Council (NHMRC) guideline recommends that children should not drink before the age of 18.
2. It is protective for teenagers if there is a household rule set by parents that they should not drink alcohol or be provided with alcohol before the age of 18.
3. It is illegal for adults to serve alcohol to children that are not their own without written or verbal consent from the child’s parent or guardian.

Between lesson 2 and 3 leaflets highlighting these messages will be sent to parents/guardians and adolescents will be encouraged to discuss the content of these leaflets with adults in their home. The manual for this intervention component can be obtained from the research team.

2. Community-Based Sales Monitoring. Role-play shoppers will be selected by an expert panel to look younger than 18 years old and asked to purchase alcohol from community outlets. Best practice involves asking for age verification for anyone who looks under the age of 25 years. Feedback letters will be sent to outlets in the form of a letter, informing them of practice in their stores. This will clarify that the strategy is not intended to entrap but to monitor practice and encourage best practice and, moreover, that repeated failure to employ best practice will result in referral to licencing authorities. This strategy has been found to reduce alcohol sales to teenagers [9]. The manual for this intervention component can be obtained from the research team.

### Study design and data collection

The study will employ a Type II, translational, longitudinal, randomised control trial to evaluate CTC. Fourteen pairs of communities (clusters) matched on socio-economic status (SES), location, and size from three Australian states (Victoria, Queensland and Western Australia) will be recruited and one of each pair will be randomised to the CTC intervention or a no-intervention control. Intervention sites can be obtained from the research team, control sites will be available after the trial is complete.

### Participants and research eligibility

**Fig. 1:**
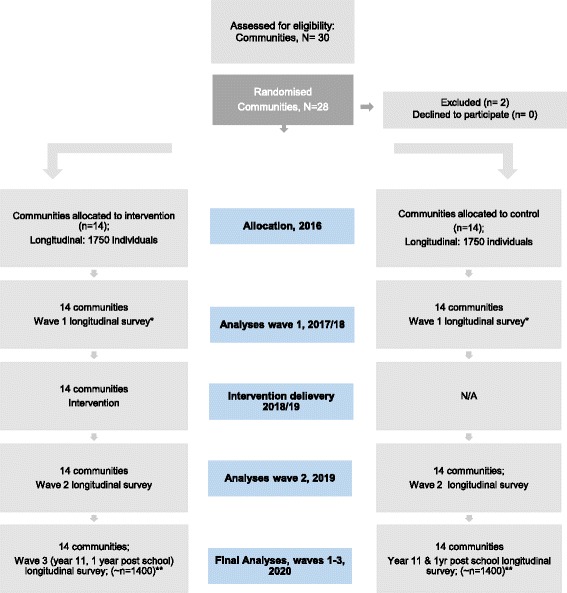
Flow chart estimating the progress of communities, schools and participants through trial. Trial registration: ACTRN12616001276448. *Wave 1 will be with either year 8 & 10, or Year 9 and 11, depending on speed in which communities work through the CTC phases, and the ease in which ethics is approved in the relevant school jurisdictions, and thus when the subsequent collection of survey data can occur. Final wave will be with year 11 students and 1 year post school. ** Approximate estimate of sample size of longitudinal cohort after 80% retention rate

The primary hypothesis will be tested using a longitudinal school survey. Three waves of data will be collected; pre-intervention (baseline), and two waves post-intervention (see Fig. 1, Flow diagram).

Two secondary schools from each of the 28 communities will be invited to participate. Informed consent of school leaders will be required for school participation and if a school declines a nearby school will be approached. The survey will include items that assess alcohol use, and other related-behaviours as well as risk and protective factors (see measures below).

Students in the participating schools will be invited to complete the survey if they satisfy the following inclusion criteria:

1. Are in the relevant (either Year 8 or 10) secondary school classroom grades (average age Grade 8 = 14 years; Grade 10 = 16 years);
2. Have signed informed consent from their parents; and
3. Have provided individual assent.

### Sample size

Previous data indicate the intraclass correlation coefficients (ICC) for alcohol consumption in the last 30 days at community and school levels were .01 and .05, respectively. We calculated the required sample with power 0.8, type I error 0.05 and design effects based on clustering effect (ICC) at community, school and classroom clustering ranging from 0.1 to 0.01. Recent Australian secondary school survey data [10] indicate rates of alcohol use in the past month were 22% in grade 8 (average age 14) and 48% in grade 10 (age 16) (combined average 35%). Assuming 65% of parents consent to their child participating, we will request informed consent from 5400 to recruit 3500 students in 2017 (wave 1). These cohorts will be resurveyed at two time points after the intervention is delivered. Assuming an 80% retention rate, the hypothesised 15% reduction can be detected with 2800 participants in the longitudinal analyses. The research team will work with communities and relevant stakeholders to recruit schools for the study. Primary outcome data will be collected in the school setting using the CTC youth survey.

### Survey measures

Survey measures will be based on validated measures of behaviours and validated risk and protective factors [11] and archival data. The survey instrument has been used in large CTC trials in the USA and Australia [12]. More information about the survey instrument can be found here (https://www.communitiesthatcare.org.au/5-phases-ctc). Example questions are provided below. Primary outcome data in control and intervention sites will be collected in the school setting using the CTC youth survey.

### Primary outcome measure

*Alcohol use in the last 30 days:* “In the past 30 days have you had more than just a few sips of an alcoholic beverage (like beer, wine, or spirits) (yes/no)?” This measure has been validated in surveys and community trials [1, 13, 14].

### Secondary outcomes

Survey data will also be used to assess a series of secondary outcomes.

*Alcohol risk and harm*: recent risky drinking: “Think back over the past 2 weeks. How many times have you had five or more alcoholic drinks in a row?”

*Alcohol use disorders* will also be investigated using questions from the Alcohol Use Disorders Identification Test (AUDIT) [15].

*Intention to drink before 18:* National Australian guidelines recommend that adolescents should avoid alcohol before the age of 18; students will, therefore, be asked about intention to use alcohol before the age of 18: “I do not intend to consume alcohol before I am 18 years of age (yes/no)?”

*Illicit drug use:* “In the past 30 days, have you: Used marijuana (pot, weed, grass)?; Used other illegal drugs (like cocaine, heroin, ecstasy, or amphetamines/speed?”

*Tobacco use:* Lifetime, past year and past 30 days assessments will be made. For example, “In your lifetime have you ever smoked cigarettes?”

*Antisocial behaviour/violence/offending:* “How many times in the past year (12 months) have you: Beaten up someone so badly that they probably needed to see a doctor or nurse?” and “Stolen something worth more than $10?”

*Secondary school engagement:* Will be measured with scales assessing school commitment, academic performance, and attendance. School completion will be based on successfully completing secondary school.

*Depression*: The short mood and feeling questionnaire (SMFQ) will be used to measure adolescent depression [16].

### Community-level archival data

School performance will also be determined using archival data from the National Assessment Program – Literacy and Numeracy (NAPLAN) data. The NAPLAN captures data for four domains (reading, writing, language conventions and numeracy). Aggregated school-level data is publicly accessible via the My School website (https://www.myschool.edu.au/). School-level performance data and school-level attendance data are also publicly accessible from the My School website.

Community level data will also include archival data on adverse events, such as alcohol-related hospital admissions; medical visits; alcohol related treatments; and police arrests for assault and related-offences, including criminal court appearances (e.g. [17]).

### Data collection in control communities

The control communities will be observed using: (1) the longitudinal school survey with 3 waves of data (see Fig. 1, flow); (2) monitoring of underage alcohol sales and (3) and archive record assessments. See schedule of enrolment and measures in Table 1.

**Table 1:** Schedule of enrolment, intervention and assessments

		STUDY PERIOD					
	Enrolment	Allocation	Post-allocation				Close-out
TIMEPOINT	*2016*	2017	*2017/18*	*2018/19*	*2019*	*2020*	*2020*
Cluster randomization	X						
Eligibility screen		X					
Informed consent		X					
Allocation		X					
INTERVENTIONS:							
* Brief communications in Schools*				X			
* Sales Monitoring*				X			
* Control*							
ASSESSMENTS:							
* Communities That Care Youth Survey*			X		X	X	X
* Community level archival data*			X		X	X	X

### Process evaluation: intervention implementation measures

The CTC framework specifies milestones and benchmarks. Benchmarks include development of a work plan and timeline for the collection of implementation goals and outcome data for each prevention program implemented. Community implementation plans for program (Phase 4 and 5) will stipulate how it will be effective programs will be implemented and monitored to ensure that five fidelity factors are met as follows:

1. Adherence: all components of the effective programs are used as stipulated in manuals; critical content is not added or removed;
2. Dosage: the required number of sessions are delivered in the correct order and over the correct time periods for each program;
3. High quality delivery: programs are delivered by qualified, expert staff;
4. Participant involvement: the programs are delivered so participants engage with the content in line with program logic models;
5. Saturation or reach: The program reaches target population.

Program specific checklists, observations, registration records, participant attendance logs, evaluation surveys, and training attendance logs will be used to assess implementation fidelity.

### Random allocation

The community sampling frame was initiated by selecting all Statistical Local Areas (SLAs) with greater than 17,000 inhabitants across the Australian States of Victoria, Queensland, and Western Australia. These SLAs were stratified into quartiles of socioeconomic disadvantage based on Socio-Economic Indexes for Areas (SEIFA) [18, 19]. Thirty eligible communities were randomly selected from SEIFA quartiles to represent state distributions in advantage/disadvantage and urban and nonurban locations. Of the 30 originally sampled SLAs, two were excluded from the present study due to their prior involvement in the CTC approach (see Fig. 1). The remaining 28 SLAs were paired based on SEIFA scores, urban or nonurban locations and size. An independent statistician using a computerised random number generator, allocated one of each pair to the intervention and control group, resulting in 14 intervention and 14 control SLAs, and was blind to allocation sequence.

### Statistical analyses

Intention-to-treat analyses will be employed. To account for within-individual variation we will employ repeated measures models with split-plot in time design matrix with binary outcome and logit link. Model parameters will be estimated using generalised estimating equations (GEEs) accounting for correlation within participants with an exchangeable working correlation matrix. For continuous secondary outcomes, GEEs with a Gaussian distribution and identity link will be implemented.

To assess the impact of the intervention on primary and secondary outcomes, time by treatment interactions will be examined in a model that includes the fixed categorical effects of assessment time, group indicator and treatment by group interaction. Trend effects due to staggered rollout of the intervention will be assed using models with a fixed effect categorical factor for year of treatment.

Clustering effects for schools and communities will be evaluated by calculating ICC and, if necessary, a series of (generalised) linear mixed models accounting for *clustering* (within participant/class/school/community). Analysts will not be blind to the communities that have been allocated to either intervention or control.

### Sensitivity analysis

Primary analyses assume the missing data are missing at random (MAR). It is thus important to assess the sensitivity of analysis under the MAR assumption to the not missing at random (NMAR) assumption. We will investigate various scenarios of non-ignorable missing data mechanisms through a sensitivity analysis based on a mixture modelling approach. Missing data will be imputed under MAR assumption first, and parameter estimates for each imputed data set will be obtained [20].

### Secondary outcome analyses

Per-protocol and subgroup analyses will be performed. Depending on sample size, subgroup analyses will examine differential effects by age, gender, community, rural areas, socio-economic status, State, and the extent of implementation and fidelity to the CTC process. Using (generalised) structural equation models, analysis of whether effects intervention processes on multiple outcomes (e.g. alcohol and drug use; violence; school completion) are mediated by changes in patterns of adolescent alcohol use will be assessed, after adjusting for other risk factors. Specified intervention mechanisms examined will include: percentage of outlets selling alcohol to perceived minors, reported exposure to brief communication resources; reduced perceptions of alcohol availability from different sources; and less favourable peer, family and community attitudes to alcohol. In addition, individual-level mediation analyses will be used to explore how any observed intervention effects on knowledge, attitudes, intention and skills account for any observed differences in reported alcohol consumption over the last 30 days.

### Economic evaluation

The economic evaluation will include both a trial-based analysis (i.e. costs and outcomes exactly as per the trial period) and a modelled analysis that will estimate the longer term economic benefits for the community. This will include cost-effectiveness, cost-utility, cost-benefit and cost-consequences analyses. It is expected that the CTC intervention will:

- Result in substantial economic benefits relative to the control communities related to reductions in each of the following:

- Alcohol use problems and harms, illicit drug use and tobacco smoking;
- Alcohol-related hospital admissions (specifically alcohol-related emergency department admissions);
- Alcohol-related violence and other offending; and
- Failure to complete secondary school.
  b. Deliver an attractive internal rate of return (IRR) with economic returns well above intervention costs from a societal perspective;
  c. Be cost-effective from a health sector perspective, with the relationship between health gains and net cost well below the decision threshold commonly used in Australia (<$50,000 per Quality-adjusted life year (QALY)).

Trial outcomes will be promoted in peer-review publications and community fora.

### Registration, ethics and data management

The Trial is registered with the Australian and New Zealand Clinical Trial Registry (http://www.anzctr.org.au/; ACTRN12616001276448). The trial team started to develop relationships with community stakeholders in January, 2016, in order to build community capacity to implement the CTC framework. As community relationships were initiated, in September, 2016, the trial was retrospectively registered. The first wave of the trial and longitudinal data was collected in February, 2017.

There are no predetermined criteria for discontinuing or modifying the trial. No anticipated adverse consequences are anticipated for trial participants. In the case of any occurrence, details will be forwarded to Deakin University’s Human Research ethics committee, in accordance with the conditions of the ethics approval for the project. The current protocol (version 1) is in keeping with the current ethics approval. Any protocol modification will be, where appropriate, referred to Deakin University’s Human Research Ethics Committee and communicated through the trial registration listed in the Australian New Zealand Clinical Trials Registry.

Data management will be primarily the responsibility of the research team based at the School of Psychology, Deakin University. Management of trial data will be in accordance with a data-management protocol, which has been developed and approved by the Project Advisory Group, and available on request. The study protocol details requirements regarding data entry, data cleaning, data back-up, secure storage and transport, and analysis. The Deakin University Human Research Ethics Committee (DUHREC) has approved the project with the expectation that data will be securely stored, and accessible only to primary researchers and statisticians through allocation of access rights. Ethics approval number from DUHREC is 2,015,261.

Confidential data on school and community contact details (e.g. phone numbers, email addresses) will be stored in a secure dataset that is not linked to survey response datasets. A trained research manager will be the only person with access to confidential participant data. The National Health and Medical Research Council (NHMRC) is the primary funder of the trial (see https://www.nhmrc.gov.au/). The NHMRC will have no role in the implementation or management of the intervention, collection, management, analysis or interpretation or the publication of the trial data.

## Discussion

The Communities that Care intervention approach has been found to be effective in the USA [2, 4–6]. This trail will test its potential to reduce alcohol use among teenagers in Australia. We expect that the present approach will be associated with significant effects on alcohol use amongst adolescents because interventions adopted within communities will be based on evidence-based practices and target specific problems identified from surveys conducted within each community [2, 3]. The present study ascribes an important role to coalitions in the management and monitoring and sustainability of interventions.

## Abbreviations

AUDIT: Alcohol Use Disorders Identification Test
CTC: Communities That Care
DUHREC: Deakin University Human Research Ethics Committee
GEE: Generalised estimating eqs.
ICC: Intraclass correlation coefficients
IRR: Internal rate of return.
MAR: Missing at random
NAPLAN: National Assessment Program – Literacy and Numeracy
NHMRC: National Health and Medical Research Council
QALY: Quality-adjusted life year
SEIFA: Socio-Economic Indexes for Areas
SES: Socioeconomic status
SLA: Statistical Local Areas
SMFQ: Short mood and feeling questionnaire
USA: United States of America
